# A Deletion in the Bovine *FANCI* Gene Compromises Fertility by Causing Fetal Death and Brachyspina

**DOI:** 10.1371/journal.pone.0043085

**Published:** 2012-08-29

**Authors:** Carole Charlier, Jorgen Steen Agerholm, Wouter Coppieters, Peter Karlskov-Mortensen, Wanbo Li, Gerben de Jong, Corinne Fasquelle, Latifa Karim, Susanna Cirera, Nadine Cambisano, Naima Ahariz, Erik Mullaart, Michel Georges, Merete Fredholm

**Affiliations:** 1 Unit of Animal Genomics, GIGA-R & Faculty of Veterinary Medicine, University of Liège (B34), Liège, Belgium; 2 Department of Large Animal Sciences, Faculty of Health and Medical Sciences, University of Copenhagen, Frederiksberg, Denmark; 3 GIGA-R Genotranscriptomics Core Facility, University of Liège (B34), Liège, Belgium; 4 Division of Genetics and Bioinformatics, Department of Animal and Veterinary Basic Sciences, Faculty of Health and Medical Sciences, University of Copenhagen, Frederiksberg, Denmark; 5 CRV BV, Arnhem, The Netherlands; Institut Jacques Monod, France

## Abstract

Fertility is one of the most important traits in dairy cattle, and has been steadily declining over the last decades. We herein use state-of-the-art genomic tools, including high-throughput SNP genotyping and next-generation sequencing, to identify a 3.3 Kb deletion in the *FANCI* gene causing the brachyspina syndrome (BS), a rare recessive genetic defect in Holstein dairy cattle. We determine that despite the very low incidence of BS (<1/100,000), carrier frequency is as high as 7.4% in the Holstein breed. We demonstrate that this apparent discrepancy is likely due to the fact that a large proportion of homozygous mutant calves die during pregnancy. We postulate that several other embryonic lethals may segregate in livestock and significantly compromise fertility, and propose a genotype-driven screening strategy to detect the corresponding deleterious mutations.

## Introduction

Fertility is one of the economically most important traits in cattle breeding. It is commonly measured using a series of metric “interval” traits (f.i. calving to first insemination, calving to last insemination ( = “days open”), calving to calving, etc.), as well as binary “non-return” (i.e. into oestrus) traits (pregnancy rate at 28, 56, … days after insemination) and stillbirth. Fertility is influenced by three individual animal components: dam (f.i. ability to resume oestrus cycle after calving, oocyte fertilizing capacity, uterine capacity), sire (f.i. semen fertilizing capacity), and offspring (f.i. developmental capacity of the embryo/fetus). The maternal component of fertility ( = “female fertility”) has received increasing attention as it has been steadily decreasing over the last twenty years (particularly in the most populous Holstein-Friesian dairy cattle population), and has become the primary cause for culling dairy cows. Female fertility is now commonly included in selection indexes (f.i. [1]). Male fertility is obviously an important trait for the artificial insemination (AI) industry, and consequently monitored very closely. The offspring's contribution to fertility is in essence not studied *per se*. Female and male fertility are characterized by low heritabilities (∼5–10%), and - with the exception of reciprocal translocations (f.i. [2]) - it has proven difficult to reliably identify QTL, let alone genes, that influence these traits. Significant correlations between genomic and realized breeding values suggest a quasi-infinitesimal architecture involving many genes with individually very small effects (f.i. [3]).

Brachyspina syndrome (BS) is a rare (<1/10^5^ birth) congenital defect that was recently described in Holstein-Friesian cattle [4]–[7]). Affected animals are characterized by severely reduced body weight, growth retardation, extensive vertebral malformations causing a significant shortening of the spine (brachyspina) and long and slender limbs. In addition, affected calves exhibit inferior brachygnatism (i.e. uneven alignment of the upper and lower teeth), as well as malformation of the inner organs, in particular the heart, kidneys and gonads. All reported cases trace back on both sire and dam side to *Sweet Haven Tradition*, a once popular sire, suggesting autosomal recessive transmission.

In this work, we use high-density SNP arrays and next generation sequencing (NGS) to identify the mutation causing BS. We make the unexpected observation that carrier frequency is as high as 7.4% in the Holstein-Friesian breed. We provide strong evidence that the discrepancy between disease incidence and carrier frequencies in this population is due – at least in part - to death of affected fetuses during pregnancy. We raise the hypothesis that other, as of yet unrecognized recessive lethal mutations likewise contribute to subfertility in cattle and propose a genotype-driven screen for their detection.

## Results

### Autozygosity-mapping positions the BS locus in a 2.5 Mb BTA21 interval

**Figure 1:**
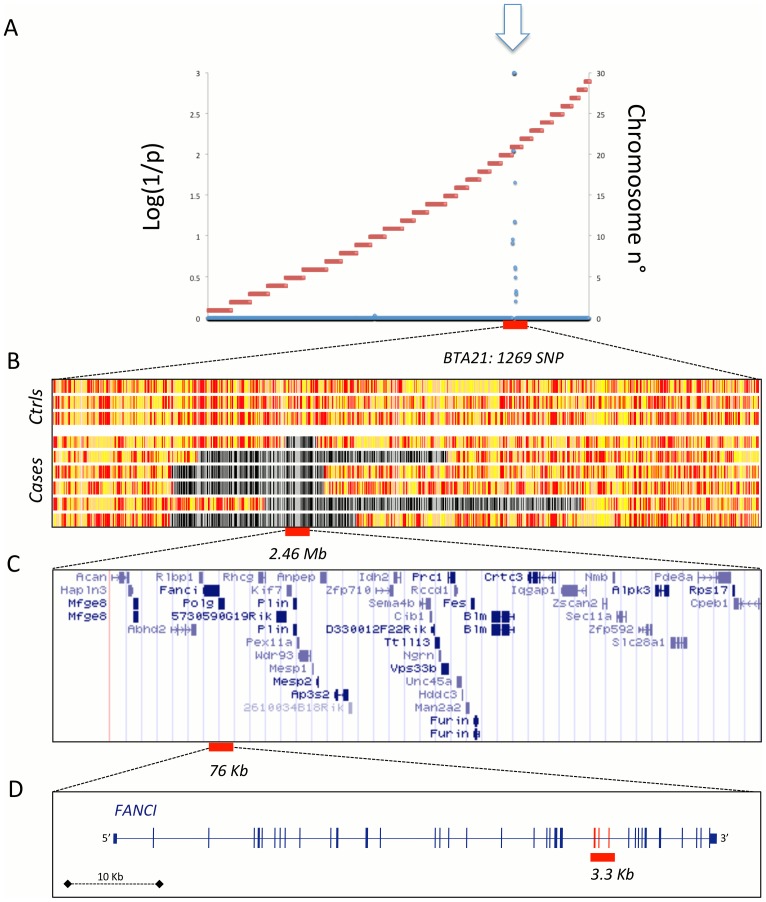
The BS locus maps to a 2.5 Mb interval on BTA21. (**A**) Autozygosity mapping of the BS locus on bovine chromosome 21 using ASSIST (Charlier et al., 2008). Blue dots measure the genome-wide probability that the six BS cases would share the observed segment of autozygosity by chance alone at each SNP position. The horizontal red lines mark the limits between adjacent chromosomes (numbered on the right Y axis). Inset: BS calf. (**B**) Genotype of three unaffected controls and six BS cases at 1,269 BTA21 SNP positions. Homozygous genotypes are shown in yellow or white, heterozygous genotypes in red. Homozygous segments encompassing the BS locus in the six cases are shown in black and white. (**C**) Gene content of the 2.46 Mb segment of autozygosity. (**D**) Map of the bovine *FANCI* gene with indication of the 3.3 Kb BS-causing deletion.

Between January 2008 and December 2009, we obtained biological material from six Holstein-Friesian calves diagnosed with BS, originating from Denmark, the Netherlands and Italy. Genomic DNA was extracted using standard procedures and genotyped using a previously described bovine 50 K SNP array [8]. Assuming that BS is inherited as an autosomal recessive defect and genetically homogeneous in Holstein-Friesian, the six cases are predicted to be homozygous for a common haplotype encompassing the causative mutation. We performed autozygosity mapping using the ASSIST program and 15 unaffected Holstein-Friesian bulls as controls, and identified a single, genome-wide significant peak (p<0.001) on chromosome 21 (BTA21) (Fig. 1A). The shared haplotype spans 2.46 Mb (bTau4.0: 20,132,767–22,588,403) encompassing 56 annotated genes (Fig. 1B, C).

### Targeted and genome-wide resequencing identifies a 3.3 Kb deletion in the FANCI gene

Seven of the 56 genes in the interval are known to cause embryonic lethality when knocked out in the mouse. We amplified the corresponding open reading frames from genomic DNA of cases and controls but did not find any obvious disruptive DNA sequence variant. We then performed targeted sequencing of the entire 2.46 Mb interval. A custom sequence capture array (Roche Nimblegen) was designed based on the bovine bTau4.0 build, and used to enrich the corresponding sequences from total genomic DNA of two affected individuals prior to paired-end sequencing (2×36 bp) on an Illumina GAIIx instrument. Resulting sequence reads were mapped to the bTau4.0 build using Mosaik (http://bioinformatics.bc.edu/marthlab). In the targeted region, the coverage of non-repetitive bases averaged 90.45 (range: 0–336) for the first sample, and 61.28 (range: 0–189) for the second, to be compared with 0.01 (range: 0–24) for the first and 0.01 (range:0–104) for the second sample outside the targeted region. The proportion of targeted non-repetitive bases with coverage <10 was 0.12 for both samples. We used the GigaBayes software (Gabor T. Marth, Boston College, http://bioinformatics.bc.edu/marthlab) to identify polymorphisms and detected 2,368 SNPs and 572 insertion-deletions for a total of 2,940 variants. One thousand thirty two of these corresponded to polymorphisms previously reported in breeds other than Holstein-Friesian (Coppieters, personal communication), and were therefore eliminated as candidate causative mutations. Of the remaining 1,908 variants, only one was coding, causing a serine to glycine substitution in the *LOC516866* gene encoding a myosin light chain kinase-like protein. This variant was not considered to be a credible candidate mutation underlying BS.

**Figure 2:**
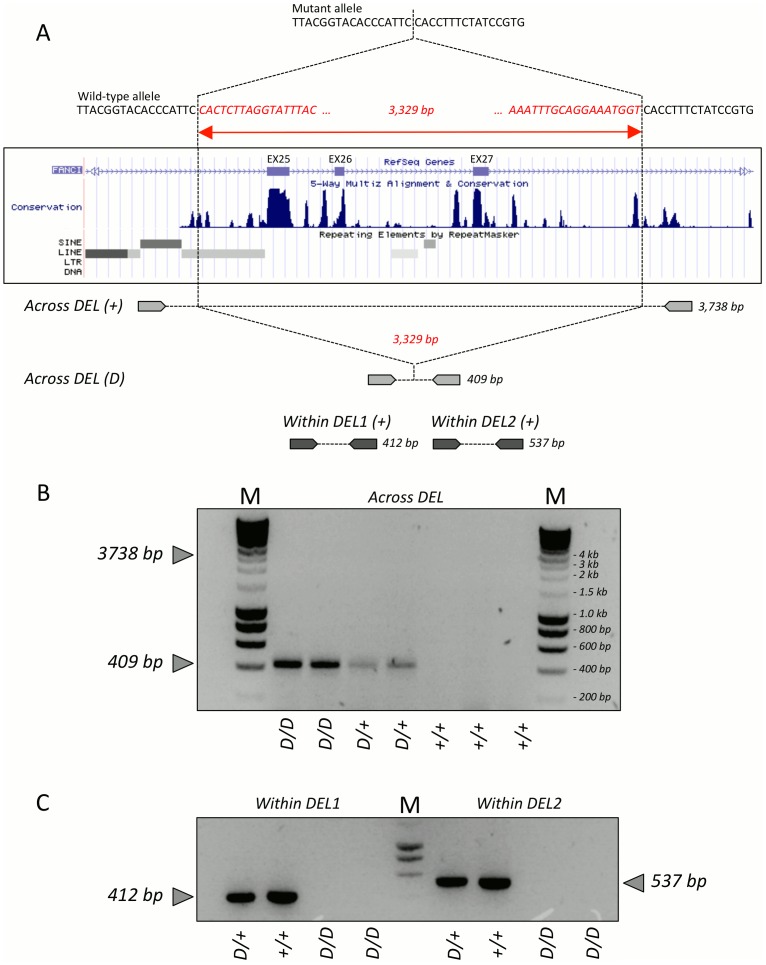
A 3.3 Kb deletion is identified in the *FANCI* gene. (**A**) Sequences and position within the *FANCI* gene of the BS deletion; position and size of amplicons used to validate the deletion. (**B&C**) PCR amplification of amplicons spanning (**B**), and residing within (**C**) the BS deletion in BS cases *(D/D)*, BS carrier *(D/+)* and homozygous wild-type *(+/+)* animals.

We then generated mate-pair libraries from self-ligated 4 to 4.5 Kb fragments of one BS case and three unrelated healthy controls, and generated ∼3.4 Gb of sequence on a Illumina GAIIx instrument for each animal. Resulting reads were mapped to the bTau4.0 build using the Burrows-Wheeler Aligner (BWA) [9], and alignments visualized with the Integrative Genomics Viewer (IGV) [10]. The achieved sequence coverage averaged 1.7× per non-repetitive base. Analysis of the reads mapping to the 2.46 Mb interval readily revealed a 3.3 Kb deletion removing exons 25–27 of the 37 composing the *FANCI* (Fanconi anemia complementation-group I) gene (Fig. 1D, Fig. 2A). The deletion was apparent from a cluster of 27 mate-pairs mapping ∼8 Kb apart on the bTau4.0 build, and from the complete absence of reads mapping to the deleted segment for the BS case, contrary to the three controls showing normal, uniform coverage in the region (Fig. S1). Retrospective analysis of the sequence reads obtained by targeted capture from affected individuals confirmed the abrupt coverage drop at the exact same location. We designed a primer pair spanning the presumed deletion, allowing productive amplification of a 409 bp product from genomic DNA of affected and carrier animals but not of unrelated unaffected controls from the same or other breeds (Fig. 2A, B). Conversely, primer pairs designed within the deletion did not yield any amplification from DNA of affected individuals compared to unaffected ones (Fig. 2A, C). Sequencing the deletion-specific amplicon defined the breakpoints, confirming a 3,329 bp deletion (Fig. 2B). Analysis of the sequence traces captured from affected individuals identified several reads bridging and confirming the breakpoint. We didn't note any obvious sequence similarity between the breakpoints.

**Figure 3:**
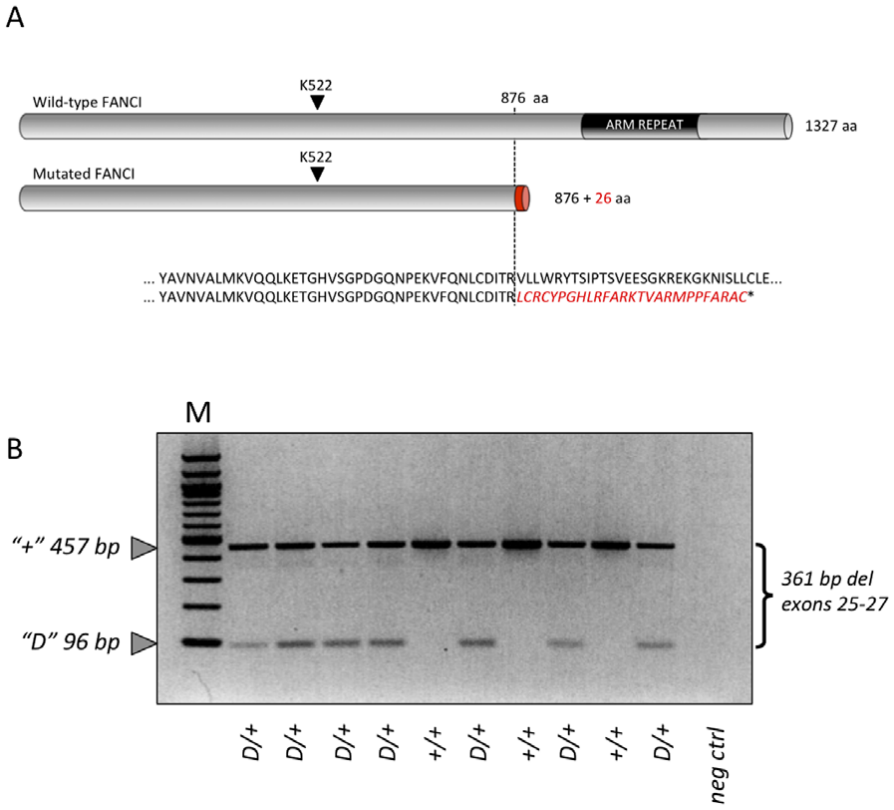
The *FANCI* deletion creates a premature stop codon responsible for mutant mRNA degradation. (**A**) Predicted effect of the BS deletion on the encoded FANCI protein. (**B**) RT-PCR products obtained from leucocyte cDNA from carrier *(D/+)* or wild-type *(+/+)* individuals using primers targeting exons 24 and 28, respectively, showing a less abundant, smaller (96 bp vs 457 bp) amplification product in *D/+* but not *+/+* animals derived from the mutant *FANCI* allele. M: molecular weight marker (100 bp ladder).

Assuming that the deletion of exons 25 to 27 results in the juxtaposition of exons 24 and 28 in the mRNA, the 3.3 Kb deletion is predicted to cause a frame-shift at amino-acid position 877, substituting the 451 carboxy-terminal amino-acids with a 26-residue long illegitimate peptide (Fig. 3A). Moreover, the ensuing stop codon in exon 28 is expected to cause nonsense mediated RNA decay (NMRD)(Fig. 3B). To examine the effect of the 3.3 Kb deletion on *FANCI* transcripts, we designed primers in exons 24 and 28 and performed RT-PCR experiments using total RNA of leucocytes from carrier and wild-type bulls. In agreement with our prediction, we amplified a smaller, 96 bp fragment from carriers but not from homozygous wild-type animals. Sequencing the 96 bp RT-PCR product indeed confirmed the juxtaposition of exons 24 and 28 in mutated *FANCI* transcripts. Despite its smaller size, the 96 bp fragment was less abundant than the 457 bp fragment corresponding to wild-type *FANCI* transcripts, supporting NMRD (Fig. 3B).

With its homologue FANCD2, the FANCI protein forms the ID complex that localizes to damage-induced chromatin foci. FANCI is essential for DNA interstrand crosslink repair. Like FANCD2, FANCI is mono-ubiquitinated by the ubiquitin ligase FA core complex, and phosphorylated by the ATM/ATR kinase (f.i. [11]). Missense, nonsense and splice-site variants in the *FANCI* gene underlie ∼2% of Fanconi anemia (FA) cases in human [11], [12]. FA patients exhibit heterogeneous symptoms, including growth retardation, skeletal abnormalities, renal, cardiac, gastrointestinal and reproductive malformations (reminiscent of BS), as well as bone marrow failure, early onset of cancer and mortality at a young age. Thus, *FANCI* qualified as a valid candidate gene.

To further support the causality of the 3.3 Kb deletion in the *FANCI* gene, we developed a genotyping assay that simultaneously interrogates the mutant and wild-type allele (cfr. M&M). As expected, all available BS cases were homozygous for the deletion. The deletion proved to be absent in a sample of 131 healthy animals representing ten breeds other than Holstein-Friesian. We then genotyped a random sample of 3,038 unaffected Dutch Holstein-Friesian animals. Carriers of the deletions accounted for 7.4% of the sample, while no animals were found to be homozygous. Assuming Hardy-Weinberg equilibrium, the absence of homozygous animals in a sample of 3,038 individuals has probability <5%. Taken together, these findings support the causality of the 3.3 Kb *FANCI* deletion.

### The 3.3 Kb Fanci deletion compromises fertility in carrier x carrier matings

We readily noticed the discrepancy between the frequency of carriers and the number of reported cases. With a carrier frequency of 7.4%, BS should represent close to 1/730 (≈0.074×0.074×0.25) births. We reasoned that this could indicate that the majority of homozygous mutant conceptuses die during pregnancy. To test this hypothesis, we compared the pregnancy failure (i.e. return in oestrus) rate at 56, 90, and 270 days post-insemination for matings between (i) a non-carrier dam and non-carrier sire, (ii) a carrier dam and non-carrier sire, (iii) a non-carrier dam and carrier sire, and (iv) a carrier dam and carrier sire. In these carrier/non-carrier status of the sire is known with certainty (from genotyping), while carrier dams are defined as daughters of carrier sires. Thus, dams defined as carrier have ∼53.7% probability to carry the BS mutation (50% probability due to the maternal grand-sire, and ∼3.7% probability due to the ungenotyped maternal grand-dam), while dams defined as non-carriers still have ∼3.7% probability to carry the BS mutation (transmitted by carrier maternal grand-dams).

**Figure 4:**
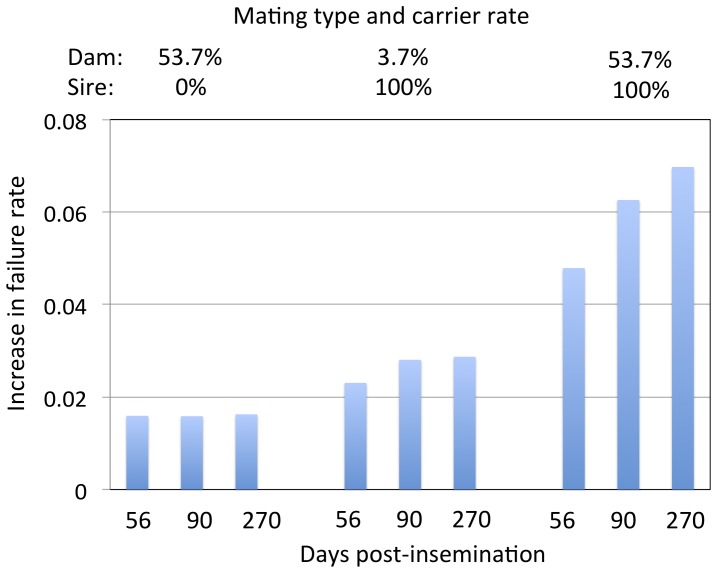
The *FANCI* deletion compromises fertility. Increased pregnancy failure rate detected as a return into oestrus at 56, 90 and 270 days post-insemination in C x NC, NC x C and C x C (over NC x NC matings). All contrasts had p-value<10^−4^.

When compared to non-carrier x non-carrier matings, failure rate at 270 days was increased by ∼7% in carrier x carrier matings (p = 5 10^−259^)(Fig. 4 and Table S1). This suggests that ∼52% of the ∼13.4% (≈0.537×0.25) homozygous mutant fetuses expected from such matings die during pregnancy. More than halve of those appear to die before day 56 of gestation, but increased mortality is still detected between days 56 and 90 as well as between days 90 and 270 of gestation (Fig. 4 and Table S1).

## Discussion

In this work, we provide strong evidence that bovine BS, a newly described congenital defect in Holstein dairy cattle, is due to a 3.3 Kb deletion encompassing exons 25 to 27 of the bovine *FANCI* gene. The deletion was the only obviously disruptive mutation found by resequencing the entire 2.46 Mb interval shared autozygous by all examined cases. Moreover, several of the lesions of BS, including growth retardation, skeletal defects and malformation of the kidneys, heart and reproductive system, are shared by patients suffering from FA.

After initial autozygosity mapping of the BS locus using a medium density 50 K SNP array, we relied on next generation sequencing to identify the causative mutation. We first performed targeted sequencing of two affect individuals using sequence capture arrays, but did not identify the causative mutation despite a sequence depth of ∼90 for the first sample, and ∼60 for the second. This was due to the fact that we could not be confident that the observed 3.3 Kb deletion in the *FANCI* gene, indeed obvious from the captured sequence reads, was due to the failure to capture the corresponding segment or to a genuine deletion. It demonstrates the need to include a healthy control in the targeted sequencing experiment. Only when analyzing the reads from the genome-wide mate-pair libraries, generated for cases and controls, did the deletion become clear.

A striking feature of BS is the discrepancy between the low incidence of the condition (∼1/10^5^ births), yet the high frequency of carrier individuals in the Holstein-Friesian population (∼7.4%). We reconciled these contradictory findings in part by providing strong evidence that at least half of BS homozygous mutant fetuses die before birth. However, this still leaves a large proportion of expected BS cases unaccounted for. Indeed, assuming a mortality of 50% of homozygous conceptuses, and given the 7.4% of carrier animals, one still expects one BS case per 1,500 newborn calves. The reasons for the remaining discrepancy remain unclear, yet we speculate that it might include (i) the fact that matings between carriers occur at a frequency <0.074×0.074 = 0.0055, as BS farmers control the rate of inbreeding by avoiding matings between closely related animals (all BS carriers trace back to *Sweet Haven Tradition*), and (ii) the fact that the statistics on failure rates at 56, 90 and 270 days do not account for inseminated cows that were culled without reported calving (“removers”). A significant proportion of these animals is known to be culled because they are not pregnant.

We noticed the significant increase in pregnancy failure rate in crosses between carrier dams and non-carrier sires as well as between non-carrier dams and carrier sires (Fig. 4 and Table S1). While in the latter cross the excess failure could be due to the fact that ∼3.7% of non-carrier dams are predicted to be misclassified (having inherited the BS mutation from an expected 7.4% of grand-dams), the excess failure rate in the former is more intriguing. We are presently examining whether this observation reflects a polygenic effect associated with the BS mutation, or whether it involves other biological mechanisms. Preliminary results point towards segregation distortion and effects on global recombination rate of the BS mutation (Charlier, unpublished observations). The relationship between these two observations and increased pregnancy failure rate in crosses involving one carrier parent are currently being examined.

Our findings strongly suggest that the main economic impact of the BS mutation is through its effect on fertility, one of the economically most important traits in cattle breeding. Similar observations were previously reported in the same breed for Complex Vertebral Malformation (CVM) [13]–[16]. The BS and CVM mutations could only be identified via the genetic analysis of a few surviving homozygous mutant offspring. Genetic defects, for which 100% of homozygous mutant conceptuses would die during early pregnancy, would essentially go unnoticed, as seen for deficiency of uridine monophosphate synthase [17]. Recent data from the 1,000 Genomes Project indicates that naturally occurring null alleles, of which a proportion is bound to be embryonic lethal, may be more common than initially suspected [18], and the same may apply in livestock. Next generation sequencing (NGS) technology offers the opportunity to detect such embryonic lethal mutations using a genotype-driven screening approach. We plan to use emerging capturing reagents for the bovine exome in combination with NGS to screen for coding variants that are predicted to be disruptive in 100–200 individuals from cattle breeds of interest. Subsequent genotyping of ∼5,000 animals for a list of candidate mutations with MAF≥0.03 should allow detection of a statistically significant depletion of homozygotes amongst healthy individuals supporting a deleterious effect possibly manifesting itself by its effect on fertility.

## Materials and Methods

### Ethics statement

Blood samples were collected from sires, cows and calves by trained veterinarians following standard procedures and relevant national guidelines. The samples were collected specifically for this study, with the full agreement of the farmers who owned the animals. According to the Ethics Commission of University of Liège, formal ethical approval is not required under these circumstances.

### Autozygosity mapping

DNA extraction and SNP genotyping using a custom-made bovine 50 K SNP array were conducted using standard procedures as previoulsy described [8]. Autozygosity mapping, including permutation testing, was conducted using the previously described ASSIST software [8].

### Targeted resequencing

Resequencing of the 2.46 Mb candidate region was performed on two BS affected animals, one Italian and one Danish calf, using a custom made sequence capture array (Roche Nimblgen) followed by sequencing. The capture array was designed based on the bovine bTau4.0 build. Repetitive regions were excluded in the design in accordance with the manufacturer's quality parameters. This resulted in an array with a capacity to capture 84.5% the target region. Twenty-two µg gDNA from each animal was fragmented to a size of 250–500 bp by nebulization. Linkers for sequencing library construction were annealed and capture was performed following the manufacture's protocol. Capture resulted in approximately 500-fold enrichment of targeted DNA and elimination of DNA from outside the target region, as evaluated by qPCR. Paired-end sequencing (2×36 bp) on captured DNA was performed on an Illumina GAIIx. Resulting sequence traces were mapped to the bTau4.0 build using the Mosaik assembler (http://bioinformatics.bc.edu/marthlab) and the GigaBayes software (Gabor T. Marth, Boston College, http://bioinformatics.bc.edu/marthlab) was used to identify DNA Sequence Variation.

### Genome wide-resequencing

One affected individual, homozygous for the defined IBD haplotype was selected as well as three unaffected unrelated individuals from other breeds. The Mate Pair Library Prep Kit v2 from Illumina was used to generate a ∼400–450 bp paired-end sequencing library from ∼4 to 4.5 kb genomic DNA fragments for each animal. Briefly, total genomic DNA was extracted and fragmented by nebulization, 4 to 4.5 kb fragments were end-repaired with biotin labeled dNTPs. After circularization, non-circularized DNA was removed by digestion. Remaining biotinylated circular DNA was fragmented and affinity purified. Purified fragments were end-repaired and ligated to Illumina Paired-End sequencing adapters. Each library was sequenced on one lane of the flow-cell of a Illumina GAIIx with the Paired-End module to generate high-quality reads (2×76 bp). Reads were mapped and analyzed with publicly available software: *Burrows-Wheeler Alignment Tool* (http://bio-bwa.sourceforge.net) and *Samtools* (http://samtools.sourceforge.net). The output files were readily uploaded in the *Integrative Genomics Viewer* (IGV, [10]) and visually scrutinized for structural variation.

### Mutation validation and definition of the deletion breakpoint

Three primer pairs were designed, one across the putative breakpoint and two within the putative deleted region. Corresponding primer pairs are listed in Table S2. They were used to amplify products from genomic DNA of homozygous cases, carriers and unaffected unrelated individuals using standard procedures. Amplicons were directly sequenced using the Big Dye terminator cycle sequencing kit (Applied Biosystems Foster City, CA). Electrophoresis of purified sequencing reactions was performed on an ABI PRISM 3730 DNA analyzer (PE Applied Biosystems, Foster City, CA). Sequence traces were aligned and compared to bovine reference using the Phred/Phrap/Consed package (www.genome.washington.edu).

### Effect of the 3.3 kb deletion on FANCI transcripts

Whole blood was collected on EDTA from carrier and control bulls, white blood cells were recovered and total RNA was extracted using the RiboPure™-Blood Kit (Ambion) following manufacturer instrcutions. The RNA was treated with TurboDNaseI (Ambion). cDNA was synthesized using Superscript™III First Strand Synthesis System for RT-PCR (Invitrogen). A portion of *FANCI* cDNA, across exons 25 to 27, was amplified using a pair of *FANCI* specific primers (Table S2). The PCR products were directly sequenced as described above.

### Developing a genotyping test for the 3.3 Kb mutation

A 5′ exonuclease assay was developed to genotype the BS deletion, using 5′-TGT TAG CCC AGC AGA GGA-3′ and 5′-ATT CTG AAT CCA CTA GAT GTC-3′ as wild-type PCR primer pair combined with 5′-GCA CAC ACC TAT CTT ACG GTA C-3′ and 5′-GGG AGA AGA ACT GAA CAG ATG G-3′ as mutant PCR primer pair, and 5′HEX-AGT CCC AGT GTG GCT AAG GAG TGA-3′IABkFQ (wild-type) and 5′FAM-CCA TTC CAC/ZEN/CTT TCT ATC CGT GTC CT-3′IABkFQ (mutant) as probes (Integrated DNA Technologies, Leuven, Belgium). Allelic discrimination reactions were carried out on an ABI7900HT instrument (Applied Biosystems, Fosters City, CA) for 40 cycles in 2.5 µl volume with a final concentration of 250 nM for each probe, 500 nM for wild-type primers, 350 nM for mutant primers, Taqman Universal PCR Master Mix 1× (Applied Biosystems, Fosters City, CA) and 10 ng of genomic DNA.

## Supporting Information

Figure S1 **Targeted and genome-wide resequencing of BS cases and controls.** (**A**) Distribution of the genomic distance separating random mate-pairs and mate-pairs flanking the BS deletion. (**B**) IGV screen captures of mate-pair reads mapping to the BTA21 20,536,086–20,541,232 chromosome interval for three unaffected controls (lanes 1–3) and a BS calve (lane 4), as well as paired-end reads obtained from captured DNA of a BS calve (lane 5).
(PDF)

Table S1 **Effects on fertility of the** ***FANCI*** **deletion.** Pregnancy failure rate detected as 100% minus non return into oestrus (NR) at 56, 90 and 270 days post-insemination in the four possible matings. The genotype probabilities of the dams are estimated from the knowledge of the genotype of their sire combined with the known frequency of the BS mutation in the general population.
(PDF)

Table S2 **Primer pairs to validate the deletion in the** ***FANCI*** **gene.**
(PDF)
