# Supplementary material for: A Deletion in the Bovine FANCI Gene Compromises Fertility by Causing Fetal Death and Brachyspina
Source: PLoS One. 2012 Aug 29;7(8):e43085. doi: 10.1371/journal.pone.0043085 (PMC3430679; doi:10.1371/journal.pone.0043085)

Figure S1: Targeted and genome-wide resequencing of BS cases and controls.

(A) Distribution of the genomic distance separating random mate-pairs and mate-pairs flanking the BS deletion. **(B)** IGV screen captures of mate-pair reads mapping to the BTA21 20,536,086 - 20,541,232 chromosome interval for three unaffected controls (lanes 1-3) and a BS calve (lane 4), as well as paired-end reads obtained from captured DNA of a BS calve (lane 5).

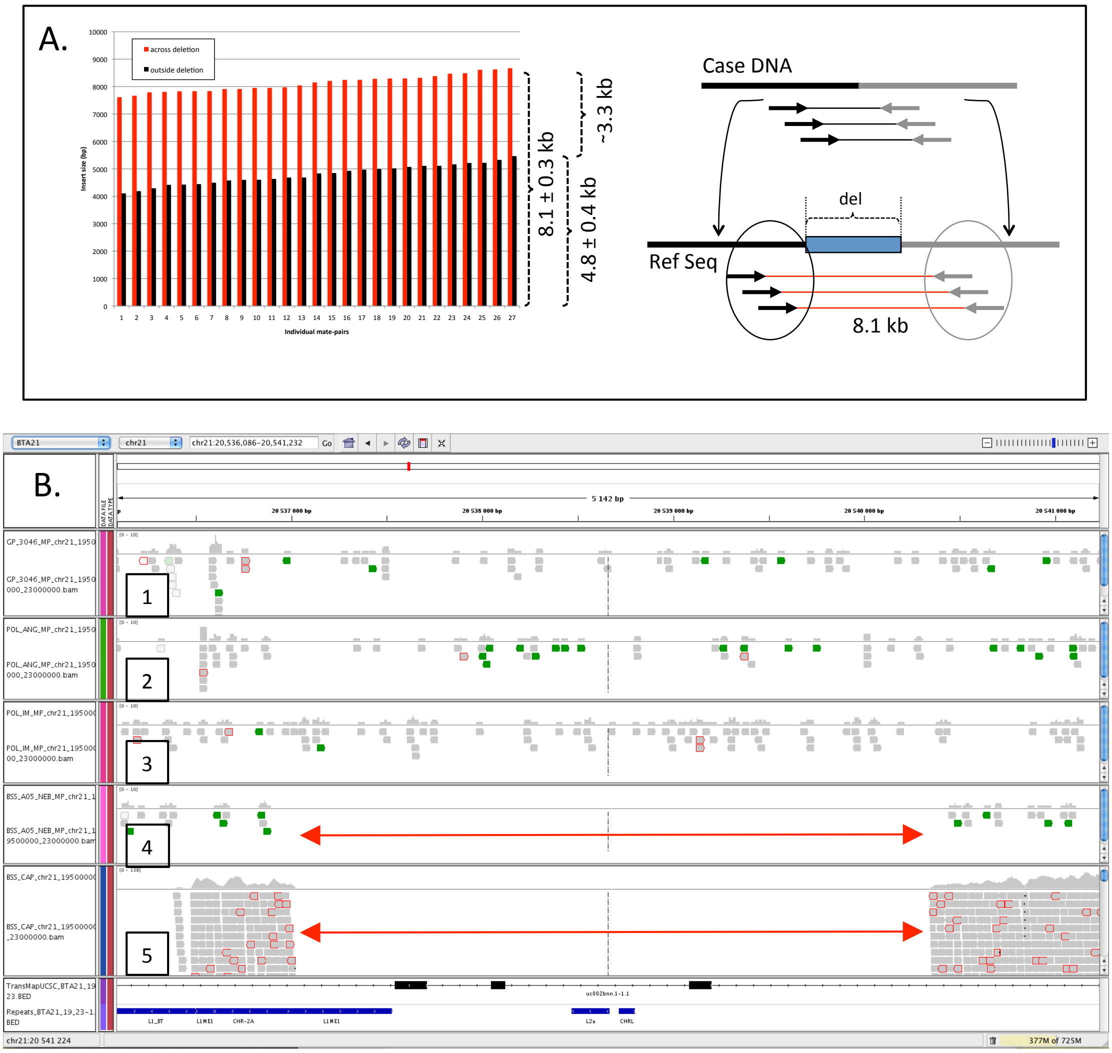

Supplement: Figure S1 — Targeted and genome-wide resequencing of BS cases and controls. (A) Distribution of the genomic distance separating random mate-pairs and mate-pairs flanking the BS deletion. (B) IGV screen captures of mate-pair reads mapping to the BTA21 20,536,086–20,541,232 chromosome interval for three unaffected controls (lanes 1–3) and a BS calve (lane 4), as well as paired-end reads obtained from captured DNA of a BS calve (lane 5). (PDF) [file pone.0043085.s001.pdf]
