# Supplementary material for: A Deletion in the Bovine FANCI Gene Compromises Fertility by Causing Fetal Death and Brachyspina
Source: PLoS One. 2012 Aug 29;7(8):e43085. doi: 10.1371/journal.pone.0043085 (PMC3430679; doi:10.1371/journal.pone.0043085)
Supplement: Table S1 — Effects on fertility of the FANCI deletion. Pregnancy failure rate detected as 100% minus non return into oestrus (NR) at 56, 90 and 270 days post-insemination in the four possible matings. The genotype probabilities of the dams are estimated from the knowledge of the genotype of their sire combined with the known frequency of the BS mutation in the general population. (PDF) [file pone.0043085.s002.pdf]

Table S1: Effects on fertility of the *FANCI* deletion.

Pregnancy failure rate detected as 100% minus non return into oestrus (NR) at 56, 90 and 270 days post-insemination in the four possible matings. The genotype probabilities of the dams are estimated from the knowledge of the genotype of their sire combined with the known frequency of the BS mutation in the general population.

Mating type	Phenotype	**WT dam (96.3% +/+)**		**Carrier dam (53.7% *D*/+)**	
**WT sire (100% +/+)**	NR56	9,391,260	34.69	1,204,592	36.29
	NR90		41.36		42.94
	NR270		45.74		47.37
**Carrier sire (100% *D*/+)**	NR56	1,025,964	37.00	112,721	39.48
	NR90		44.16		47.61
	NR270		48.61		52.71
		Total	Failure (%)	Total	Failure (%)
