# Supplementary material for: A Deletion in the Bovine FANCI Gene Compromises Fertility by Causing Fetal Death and Brachyspina
Source: PLoS One. 2012 Aug 29;7(8):e43085. doi: 10.1371/journal.pone.0043085 (PMC3430679; doi:10.1371/journal.pone.0043085)
Supplement: Table S2 — Primer pairs to validate the deletion in the FANCI gene. (PDF) [file pone.0043085.s003.pdf]

Table S2: Primer pairs to validate the deletion in the *FANCI* gene.

Primer name	Primer sequence 5’-3’	Product size *D allele*	Product size *+ allele*	Material
AcrossDEL_UP1	GCTCAAGTAGTTAGTTGCTCCACTG	409 bp	(3738 bp)	gDNA
AcrossDEL_DN1	ATAAATAAATAAAGCAGGATGCTGAAA			
WithinDEL_UP1	TCACAAAAGGGTAGGAGACTACCTG	/	537 bp	gDNA
WithinDEL_DN1	GCTTATTGTTTACCCTTGACAGTGG			
WithinDEL_UP2	ACTGGATTCCATTTAACCACAGATG	/	412 bp	gDNA
WithinDEL_DN2	ATGCATTACCTTTCATTCTCAGAGC			
Exon24_UP	GCCAAAACCCAGAGAAGGTC	96 bp	457 bp	cDNA
Exon28_DN	CAACTGTTTTCCGTGCAAAT			
